# “Together We Stand”: A Pilot Study Exploring the Feasibility, Acceptability, and Preliminary Effects of a Family-Based Psychoeducational Intervention for Patients on Hemodialysis and Their Family Caregivers

**DOI:** 10.3390/healthcare9111585

**Published:** 2021-11-19

**Authors:** Helena Sousa, Oscar Ribeiro, Constança Paúl, Elísio Costa, Roberta Frontini, Vasco Miranda, Jaime Oliveira, Fernando Ribeiro, Daniela Figueiredo

**Affiliations:** 1 Center for Health Technology and Services Research (CINTESIS.UA), Department of Education and Psychology, University of Aveiro, 3810-193 Aveiro, Portugal; helena.sousa@ua.pt (H.S.); oribeiro@ua.pt (O.R.); jaime.oliveira@ua.pt (J.O.); 2 Center for Health Technology and Services Research (CINTESIS.UA), Institute of Biomedical Sciences Abel Salazar, University of Porto, Rua Jorge de Viterbo Ferreira 228, 4050-313 Porto, Portugal; paul@icbas.up.pt; 3 Research Unit on Applied Molecular Biosciences (UCIBIO—REQUIMTE), Faculty of Pharmacy, University of Porto, Rua Jorge de Viterbo Ferreira 228, 4050-313 Porto, Portugal; emcosta@ff.up.pt; 4 Center for Innovative Care and Health Technology (ciTechCare), Polytechnic Institute of Leiria, 2410-541 Leiria, Portugal; roberta_frontini@hotmail.com; 5 NephroCare Maia, 4470-235 Maia, Portugal; vasco.miranda@outlook.com; 6 Campus Universitário de Santiago, Institute for Biomedicine (iBiMED), School of Health Sciences, University of Aveiro, 3810-193 Aveiro, Portugal; fernando.ribeiro@ua.pt; 7 Center for Health Technology and Services Research (CINTESIS.UA), Campus Universitário de Santiago, School of Health Sciences, University of Aveiro, 3810-193 Aveiro, Portugal

**Keywords:** chronic kidney disease, dialysis, family-based, psychoeducation, feasibility study

## Abstract

This pilot study aimed to assess the feasibility, acceptability, and preliminary effects of a family-based psychoeducational intervention for patients undergoing hemodialysis (HD) and their family members. This was a single-group (six dyads), six-week, pre–post pilot study, delivered in a multifamily group format. Feasibility was based on screening, eligibility, content, retention, completion, and intervention adherence rates. Acceptability was assessed at post-intervention through a focus group interview. Self-reported anxiety and depression and patients’ inter-dialytic weight gain (IDWG) were also measured. The screening (93.5%), retention (85.7%), and completion (100%) rates were satisfactory, whereas eligibility (22.8%), consent (18.4%), and intervention adherence (range: 16.7–50%) rates were the most critical. Findings showed that participants appreciated the intervention and perceived several educational and emotional benefits. The results from the Wilcoxon Signed-Rank Test showed that a significant decrease in anxiety symptoms (*p* = 0.025, *r* = 0.646) was found, which was followed by medium to large within-group effect sizes for changes in depression symptoms (*p* = 0.261, *r* = 0.325) and patients’ IDWG (*p* = 0.248, *r* = 0.472), respectively. Overall, the results indicated that this family-based psychoeducational intervention is likely to be feasible, acceptable, and effective for patients undergoing HD and their family caregivers; nonetheless, further considerations are needed on how to make the intervention more practical and easily implemented in routine dialysis care before proceeding to large-scale trials.

## 1. Introduction

End-stage renal disease (ESRD) is a life-threatening condition in which the kidneys permanently fail to function, requiring patients to undergo some form of renal replacement therapy (RRT) to survive [1]. Worldwide, the prevalence of ESRD is increasing, especially in individuals aged 65 years old and over, which is mainly fueled by other age-related chronic conditions such as diabetes and cardiovascular disease [2,3].

Hemodialysis (HD) is the most common form of RRT conducted in dialysis units, usually 3–4 times a week, for 4–5 h each session [4]. Along with attending dialysis sessions, patients must adhere to a complex regime of restrictions to fluid intake, dietary management, medication intake, vascular access care, and exercise training [5,6].

Patients have also to deal with the psychosocial consequences of ESRD and HD treatments, such as psychosocial distress and reduced quality of life [7,8]. For instance, the rate of psychiatric conditions, such as depression, in patients with ESRD is considerably higher than in populations with other chronic conditions such as cancer and congestive heart failure [9]. Furthermore, depression in ESRD has been related to poor adherence [10]. It is also well known that non-adherence to HD requirements can result in poor clinical outcomes (e.g., bone demineralization, clots in the access site, electrolyte imbalances, pulmonary congestion, heart failure) and decrease survival [11,12].

Family support has been evidenced as an important predictor of adherence among patients with ESRD [13] and is associated with improved survival and quality of life [14,15,16,17]. However, providing support to patients undergoing HD can be a burdensome experience, which is likely to involve social isolation, sleep problems, and distress symptoms [18,19,20]. Despite the profound changes for both patients and close family members, traditional renal rehabilitation programs tend to focus on the education of the patient, who receives information and skills training related to nutritional counseling, lifestyle modification, kidney disease pathology and treatment, and self-management (e.g., weight management, medication adherence, smoking cession) [21,22]. Psychosocial support is scarce, and the impacts of ESRD and treatments on family life are often superficially considered or even neglected.

To overcome these limitations, the past few years have witnessed an increasing development of family-based psychoeducational interventions as an essential component of effective treatment across a range of chronic conditions such as cancer [23], stroke [24], or respiratory diseases [25,26]. These interventions provide families with health education and psychosocial support to foster coping and adaptation, giving concrete guidelines for crisis management, problem solving, and stress reduction [27]. Within a family-oriented approach, the educational, relational, and emotional needs of the family system are emphasized. Recent meta-analytic findings on the effectiveness of family-oriented psychosocial interventions across different adult chronic diseases evidenced that these were more effective for the patients and family members’ physical and mental health outcomes than patient-focused intervention or usual medical care [28,29,30,31].

Despite the growing recognition of the benefits of family-based interventions as a component of comprehensive treatment among adults with chronic conditions, and the specific recommendations to develop them in ESRD [22], interventions to improve ESRD/HD management and adjustment remain patient-focused and, to the best of our knowledge, there is no objective evidence to sustain such orientation. Therefore, the primary goal of this pilot study was to assess the feasibility and acceptability of a family-based psychoeducational intervention for patients with end-stage renal disease undergoing HD and their family members. The secondary aim was to explore its preliminary effects on psychological distress and patients’ weight and fluid control. As the research is novel and there are uncertainties about the operational feasibility of the intervention and its acceptability among ESRD patients and families, a pilot study is highly recommended to provide useful information for the planning and justification of subsequent randomized controlled trials [32,33,34].

## 2. Methods

### 2.1. Design and Setting

This was a single-group, six-week, pre–post pilot study conducted with six dyads totaling 12 participants (six patients and six family members), who were recruited from one dialysis center in Portugal. Recruitment took place between September and October 2019. The multifamily group intervention was carried out between 26 October and 30 November 2019.

### 2.2. Participants

To be included, patients had to be adults (aged 18 years or older) diagnosed with ESRD and undergoing HD for a minimum period of three months. Patients were excluded if (i) they returned to dialysis after kidney transplant failure, (ii) had other comorbidities, such as cancer (regardless of cancer stage and/or location) or an infectious disease, and (iii) had severe mobility (e.g., amputation) and sensorial impairments (e.g., auditory or visual) that would hamper participation in the study. Family members had to be adults and the patients’ main providers of support, care, and assistance. Both patients and their family members were excluded if one of them was receiving some form of psychotherapeutic intervention and if presenting neuropsychiatric conditions and/or inability to understand and engage in the study. Finally, to be included, both parties had to understand the purpose of the study and agree to voluntary participation.

### 2.3. Recruitment

Two researchers assessed patients to determine their eligibility for the study, according to the aforementioned criteria. Those patients who were eligible were approached. Then, a researcher explained what the intervention involved and asked for willingness to participate. If the patient gave verbal consent, permission was asked to telephonically contact their main care provider in order to assess their eligibility and willingness to participate in the study. One week before the intervention, written consent was obtained from both parties. The reasons for refusing to participate were recorded by the researchers.

### 2.4. Intervention

The intervention design was informed by a comprehensive literature review on ESRD [5,35,36,37,38], evidence on family-based interventions in different chronic conditions [25,26], the researchers’ experience, and the results of interviews with HD patients and family caregivers. In fact, there are few systematic data available regarding individuals and families’ educational and supportive needs in ESRD. Acknowledging the patients’ and their family caregivers’ perspectives and opinions has been recognized as a priority to conduct research that is relevant, adequate, effective, and meaningful [39,40]. Thus, interviews with patients, family members, and patient–family dyads were conducted before the pilot study to gain an in-depth understanding of their perceived needs, expectations about the intervention, and potential facilitators and constraints to participation. The findings revealed that patients on HD and their family members congruently expected that a family-based intervention would facilitate access to information about ESRD and hemodialysis demands (e.g., fistula care, nutritional recommendations, the performance of physical activity, and availability of social, community, and financial support), increase emotional support, improve coping skills and communication with healthcare professionals, and strengthen family involvement in the disease [41,42,43,44]. Moreover, previous research has suggested that family psychoeducation usually includes the provision of information about treatments, symptom management, and community resources; skills training to respond to disease-related problems; and problem-solving and emotional-management strategies for coping with the disease demands. The rationale is based on the relevance of practical information, social support, and problem-solving assistance through the predictably stressful moments which can be anticipated in the future course of chronic disease [27].

Therefore, the intervention comprised six weekly sessions of 120 min, which were delivered in a multifamily group format. The group format was chosen as it decreases family isolation and promotes mutual support, sharing of experiences, and the exchange of useful ideas among families struggling with similar challenges [27]. Each session comprised an educative and a supportive component. Education aimed to provide information about ESRD and HD treatments, increase family skills to adjust and manage the disease, and promote strategies for managing the diet, fluid intake, exercise, weight control, and vascular access care. Psychosocial support was intended to help the family manage the emotional demands of living with ESRD and HD, develop communication skills to interact with healthcare professionals, and promote a sense of family identity and cohesion.

The sessions were conducted by a multidisciplinary team (psychologist, nurse, physician) and coordinated by two clinical psychologists with relevant experience in the development of psychoeducational interventions in chronic disease settings. These professionals assumed the role of facilitators by supporting participants in their doubts, encouraging them to share experiences, normalizing emotions, and assuming an empathic communication. Several active-learning methods were implemented, such as group discussions, role playing, brainstorming, and educational quizzes. Each session was held at the dialysis center where recruitment took place. The content of the intervention program (per session) is presented in Table 1.

### 2.5. Primary Outcome Measures

#### 2.5.1. Feasibility

The feasibility of the intervention was assessed based on the screening rate, eligibility rate and reasons for exclusion, content rate and reasons for not participating in the study, retention rate, completion rate, adherence rate, and acceptability of the intervention.

#### 2.5.2. Screening Rate

The screening rate was defined as the number of patients with ESRD who were assessed for eligibility by two researchers based on the aforementioned inclusion/exclusion criteria.

#### 2.5.3. Eligibility Rate and Reasons for Exclusion

The eligibility rate represents the number of screened patients who met the inclusion criteria; it was calculated by dividing the number of patients who met the inclusion criteria by the number of patients undergoing HD at the recruitment site.

#### 2.5.4. Consent Rate and Reasons for Not Participating in the Study

The consent rate was calculated by dividing the number of patients who verbally consented to participate by the number of patients who were eligible for the study. The consent rate of family members depended on the patient’s willingness and verbal agreement to participate in the study. The reasons for non-participation were recorded by the researchers.

#### 2.5.5. Retention Rate

The retention rate was defined as the number of patients and family members who remained in the study, i.e., the number of participants who did not formally drop out.

#### 2.5.6. Completion Rate

The completion rate was defined by the number of participants who completed the self-reported measures. Completion rates were calculated at baseline and T1 (two weeks after the end of the intervention).

#### 2.5.7. Intervention Adherence

The intervention adherence rate was measured by summing the total number of sessions attended by the participants. Reasons for not attending any of the sessions were recorded by the researchers.

#### 2.5.8. Acceptability

Acceptability is defined as a multifaceted construct that assesses the extent to which users of an intervention consider it appropriate and the degree to which it meets their needs [45]. In the current study, acceptability was assessed at post-intervention through a focus-group interview. This method has been recommended to assess the acceptability of healthcare interventions, as the analysis may reveal aspects to modify before proceeding to a large-scale trial [45]. To reduce any influence on participants’ responses, the interview was conducted eight weeks after the end of the intervention by two researchers who were not involved in its delivery. A semi-structured guide was used to explore participants’ perspectives on the impacts, benefits, and disadvantages of the intervention; barriers and facilitators for participation; and suggestions about its contents, structure, and organization. The focus group interview lasted approximately 111 min.

### 2.6. Secondary Outcome Measures

#### 2.6.1. Symptoms of Anxiety and Depression

To assess the presence of symptoms of anxiety and depression, participants completed the Hospital Anxiety and Depression Scale (HADS) [46,47]. This instrument has 14 items and is divided into two subscales: seven items that assess the presence of anxiety symptoms and seven items that assess the presence of depression symptoms in the last week, using a 4-point Likert scale ranging from 0 to 3. Scores range from 0 to 21 for each subscale. Internal consistency values in this study sample ranged from 0.714 to 0.849 for anxiety, and from 0.779 and 0.828 for the depression subscale, which is an indicator of good reliability. This self-report measure was administrated at baseline and post-intervention.

#### 2.6.2. Interdialytic Weight Gain (IDWG)

IDWG has been the most used indicator to assess fluid control in patients undergoing HD and has also been suggested as a potential marker of the patients’ nutritional status [48,49]. A monthly average (12 consecutive sessions) of the IDWG values was calculated, both before and after the intervention. Data were retrieved from patients’ medical records. According to the National Kidney Foundation Kidney Disease Outcomes Quality Initiative (KDOQI) clinical guidelines [50], for patients on HD three times a week, the IDWG should be less than 4.0–4.5% of the patients’ dry weight.

### 2.7. Quantitative Data Analysis

Descriptive statistics were calculated for all sociodemographic (e.g., age, gender, marital status, kinship) and clinical (e.g., length of time on dialysis) variables. To analyze the pre and post-intervention within-group differences, the Wilcoxon Signed-Rank Test was performed. A value of *p* < 0.05 was used to determine statistical significance. All statistics were computed using the Statistical Package for the Social Sciences (SPSS) (IBM Corp. Released 2021. IBM SPSS Statistics for Windows, Version 28.0. Armonk, NY: IBM Corp.). Effect sizes were calculated (Cohen’s r) and interpreted as follows: 0.12 = small effect, 0.24 = medium effect, 0.41 = large effect [51].

### 2.8. Qualitative Data Analysis

The focus group interview was audio-recorded, transcribed verbatim, and analyzed by two independent researchers using content analysis. Data were categorized following Braun and Clark’s recommendations [52]. First, the transcript was read and re-read to gain a sense of the whole dataset. Second, researchers highlighted expressions and sentences that captured the information related to the research questions. Finally, the information was grouped in areas expressing similar concepts and converted into categories by systematically gathering relevant quotes. Then, these initial themes were compared and reviewed to reach a consensus. A third author was consulted in case of disagreement. The final decision was based on the richness of each theme/subtheme. To increase trustworthiness, confirmability (i.e., the objectivity of the researcher), and reflexivity, three authors (authors’ names removed for anonymity) were involved in data analysis; therefore, the final subthemes were the product of an interactive process of critical dialogues and discussion. All the research team members had experience in working with patients and families living with chronic diseases, but none had personally experienced an ESRD diagnosis.

### 2.9. Ethical Considerations

The study received full approval from the Ethical Committee of the Fresenius Medical Care-Portugal (Reference number 03/2019). All procedures performed in this study followed the 1964 Helsinki Declaration and its later amendments. Written consent forms were obtained before data collection and study participation. Confidentiality was guaranteed by assigning each patient a numerical code.

## 3. Results

### 3.1. Participants’ Characteristics

Participants’ sociodemographic and clinical characteristics are presented in Table 2. Patients with ESRD (*n* = 6) had a mean age of 65.3 ± 13.3 years old and presented no gender imbalance (three men and three women). Most of them were married (*n* = 5) and retired (*n* = 5). Patients were on HD three times a week for an average of 50.7 ± 59.2 months. Family members (*n* = 6) had a mean age of 47.3 ± 15.6 years old and were all women.

### 3.2. Primary Outcomes

#### 3.2.1. Screening Rate

The screening rate was 93.5%. One hundred and sixty-seven patients were receiving HD in the recruitment site over four months (from July to October 2019). One hundred and fifty-six patients were assessed for eligibility.

#### 3.2.2. Eligibility Rate and Reasons for Exclusion

The eligibility rate was 22.8%, as 38 patients (out of 167) met the eligibility criteria. The remaining (*n* = 118) were excluded due to one of the following exclusion criteria: having a neuropsychiatric disorder (*n* = 36); lack of mobility or presence of sensorial impairment (*n* = 22); receiving anticancer treatment (*n* = 16); kidney transplant failure (*n* = 14); not being able to read (*n* = 8); having an infectious disease (*n* = 7); not having a family carer (*n* = 6); receiving psychotherapeutic intervention (*n* = 5); the main care provider was receiving psychotherapeutic intervention (*n* = 3); and being on HD for less than three months (*n* = 1).

#### 3.2.3. Consent Rate and Reasons for Not Participating in the Study

The consent rate was 34.2%, as 13 patients (out of 38) gave their verbal consent. The remaining patients (*n* = 25) refused to participate due to: (i) lack of transport to the dialysis unit on the days of the intervention (*n* = 8), (ii) did not wish to be involved in more “disease-related activities” (*n* = 6), (iii) lack of availability of the family member (*n* = 6), and (iv) perceived lack of need (*n* = 5). Family members’ consent rate was dependent on the patient’s verbal consent and willingness to participate in the intervention. Therefore, 13 family members were contacted; three refused to participate due to lack of availability or interest. The consent rate for family members was 76.9%.

The consent rate was recalculated one week before the intervention. At this stage, six patients were no longer interested in participating for the following reasons: (i) the family member was no longer interested or available (*n* = 3), (ii) medical reasons (*n* = 2), and (iii) transportation issues (*n* = 1). The final consent rate was 18.4%.

To this end, seven dyads (14 participants, i.e., seven patients and seven family members) gave written consent and were included in the intervention.

#### 3.2.4. Retention Rate

The retention rate was 85.7% as two (one dyad) out of the 14 participants formally dropped out of the study after the first session of the intervention program. The reason presented was the caregiver’s work responsibilities.

#### 3.2.5. Completion Rate

The completion rate was 100% for both assessment points for all self-report measures.

#### 3.2.6. Intervention Adherence

Two participants (16.7%) completed the six sessions, six participants (50%) attended five sessions, and four participants (33.3%) completed four sessions. The main reasons for missing the intervention sessions were the family members’ unavailability due to work and family responsibilities, participation in other sociocultural activities, and sudden worsening of the patient’s clinical condition.

#### 3.2.7. Acceptability

Overall findings from the focus group interview revealed that participants congruently shared positive experiences about the intervention. Four themes were identified in the content analysis: (i) perceived educational and emotional benefits; (ii) downsides of the intervention; (iii) barriers and facilitators for participation; and (iv) suggestions about the intervention’s content, structure, and organization.

Regarding benefits, participants reported that the intervention helped to improve their knowledge regarding ESRD and HD, dietary restrictions, vascular access care, and the importance of physical exercise, while also developing new skills regarding problem solving and emotional regulation: “*We have acquired new knowledge about our disease and HD. There’s nothing like having a healthcare professional explaining it to us in person; even in relation to our emotions and how to deal with them, how to deal with our fears, because this disease is worrying and our family also struggles with it”*. Concomitantly, the intervention helped to reframe ESRD and realize that the negative impacts of HD (e.g., disease-related problems and the disruption of family projects) could be managed and overcome: *“Along with learning, what I had the most benefit from was the new perspective on the disease. (…) This [intervention] reminded us of different situations and helped us to explore other hypotheses to deal with our problems. It made us see that there is a before, a present, but also a future beyond our illness”.* The presence of family members was perceived as a benefit, as they also need support to manage ESRD/HD care demands. In addition, pamphlets and infographics were described as crucial to facilitate the assimilation of information acquired during the sessions. Only one main downside was mentioned, as some participants felt that the themes could have been further explored and that this was not possible due to the reduced number of sessions: *“Sometimes I felt that sessions were interrupted and disconnected. Perhaps if more sessions had been performed, some themes could have been further explored, because two hours [per session] is not enough”.*

Regarding barriers and facilitators for participation, participants pointed out that it was difficult to attend intervention sessions, especially on days when the patient received treatment: *“My mother was on dialysis in the morning, then came home and then had to leave again to come here [to the dialysis center to attend the intervention session]. (...) It’s complicated for her, because she’s very tired”.* In turn, the support received by the psychologists who facilitated the group dynamics and the bond established between the participants, which favored mutual support and sharing, were identified as facilitators: “*I think we all felt that we were all in this together, that it’s not just us [the dyad], but it’s all of us. This is our problem. Realizing that was very important”.*

Participants also made some key suggestions for the development of future interventions. Most of them agreed that additional information needed to be delivered on the social benefits available to patients and informal caregivers, on dietary restrictions and how to manage them creatively, and on kidney transplantation: *“Many of us talked about our concerns about the transplant and this was not part of the program. (...) I think this subject should have been discussed more specifically, especially for patients who are on the transplant list”.* Regarding the structure and organization of the intervention program, some suggested that the sessions should be longer, as there was no consensus on increasing their number. In this sense, more time per session would allow the deepening of topics on which the need for more information was perceived, as well as the inclusion of new themes to be addressed: *“I think more session time, for example, instead of two hours, it should be two and a half hours. This would be important to further explore each topic and include new ones”.* Participants also agreed that this type of intervention—with an educational and support component—would be essential for the patient starting dialysis since, at that time, both the patient and family caregivers have to cope with numerous treatment-related demands and life changes: *“I needed this when I started the whole treatment process. If I had had this intervention before, it would have been even better, because this disease is a big puzzle to disassemble, especially in the beginning”.*

In the end, participants congruently stated that they would recommend this type of intervention to other patients and their family caregivers, as they found the program very useful, enlightening, and supportive: *“If I find out about a kidney patient or family member who needs support, I will be the first to refer them to this intervention. It is very important to be with healthcare professionals who can transmit knowledge and make them feel that they have someone on their side”.*

### 3.3. Secondary Outcomes

#### Preliminary Effects

The Wilcoxon Signed-Ranked Test (Table 3) showed a statistically significant decrease in anxiety symptoms, with a large effect size (*p* = 0.025, *r* = 0.646). Symptoms of depression also decreased after the intervention, but the results did not reach statistical significance; however, a medium effect size was found (*p* = 0.261, *r* = 0.325). Patients’ IDWG decreased after the intervention; the results did not reach statistical significance, but a large effect size was evidenced (*p* = 0.248, *r* = 0.472).

## 4. Discussion

Family is among the most important resources for patients on HD, assisting them with dietary and fluid management, reinforcing to attend dialysis sessions, reminding to take medication, providing emotional support, assisting with decision making, encouraging patient’s self-care, providing transportation, and communicating with health professionals on behalf of the patients [17,18,19]. Despite this, the effectiveness of family-based interventions remains largely unknown in the context of ESRD. To the best of our knowledge, this was the first pilot study exploring the feasibility, acceptability, and preliminary effects of a family-based psychoeducational intervention for patients undergoing HD and their closest relatives. The overall findings suggested that this intervention is likely to be feasible, acceptable, and effective; however, some aspects need to be carefully considered before proceeding to large-scale trials.

In the present study, a large number of patients were screened for eligibility (93.5%), suggesting that those recruited are likely to be representative of the target population [34]. This excellent screening rate was followed by a modest eligibility rate (22.8%), which has already been reported in previous studies aiming to explore the feasibility of psychological interventions for patients undergoing HD [53]. Severe cognitive impairment (23.1%), limited mobility (14.1%), and cancer diagnosis (10.3%) were the most common reasons for the exclusion of participants from the current study. Previous research has already reported that the prevalence of cognitive impairment among patients with ESRD ranges from 16 to 38% [54]. In fact, patients receiving dialysis were found to have more cognitive deficits than the general population in domains such as learning, memory, complex attention, executive function, language, and perceptual-motor function [55,56,57]. Risk factors such as advanced age, uremic status, cerebrovascular diseases, stroke, depression, intradialytic hypotension, acute hemodynamic changes, and inflammation caused by dialysis itself have been associated with acute cognitive deterioration in patients with renal failure [58,59,60,61]. Chronic kidney disease has also been considered a model of accelerated aging, leading to reduced cognitive and physical functioning, which increases frailty and the risk of loss of independence [56,57], compromising participation in psychoeducational interventions. Therefore, studies are needed to design and test the effectiveness of interventions to specifically support patients with cognitive impairment undergoing dialysis as well as their family caregivers who may be at increased risk of depression and burden [62].

Likewise, the consent rate was also suboptimal (18.4%) compared to interventions involving patients with cancer [63]. Understanding reasons for non-participation is of paramount importance, as this information can be used to improve consent and minimize imprecision in large-scale trials [34]. In the current study, not having transport to the dialysis center on the days of the intervention (24%) was pointed out as the main reason for denying participation. It is noteworthy that patients are guaranteed free transport to the dialysis center to receive HD treatment; however, this does not include intervention sessions of this particular study, which can represent an additional financial cost. Although raising ethical concerns that need to be carefully considered (e.g., participants motivation), the use of monetary compensation can facilitate additional traveling to the dialysis center for intervention days which, in turn, can increase consent rates [64].

Once consented, the retention rate (85.7%) of the participants was satisfactory [65], while completion was excellent (100%). Low levels of adherence are the main critical component of this study, as only two participants (16.7%) completed the entire intervention program, six (50%) attended five sessions, and four (33.3%) attended only four of the six sessions. Since non-adherence increases the risk of rejecting interventions that might actually be effective [34], it is crucial to assess its acceptability. In this sense, the qualitative findings of the focus group interview revealed that participants congruently appreciated the intervention and perceived several educational and emotional benefits. Increased disease and treatment-related knowledge, opportunities for mutual support and sharing, emotional validation, training of coping skills, and greater hope for the future, despite ESRD demands, were some of the main positive aspects identified. However, some participants mentioned the time and effort needed to attend the intervention as barriers for participation, which has already been reported in clinical trials designed to support patients undergoing HD [53]. With the strict regime of attendance to this treatment, it is possible that for some patients, an additional visit to the dialysis center may increase the treatment burden; therefore, future studies are needed to explore alternative ways of delivering the intervention without a considerable impact on (the already disrupted) family routines. Online interventions can be a viable way to provide psychoeducational support for patients with ESRD and their family caregivers. This modality can also help to increase the consent rates, as it can be attended at home and does not involve transportation expenses. A recent feasibility study of technology-assisted cognitive–behavioral therapy for patients on HD showed promising effects, good adherence rates (80%), and high acceptability and satisfaction with the video-conferencing sessions [66]. Since these interventions have been patient-oriented, studies are needed to better understand the feasibility and acceptability of family-based psychoeducational online interventions in ESRD.

This study also aimed to investigate the preliminary effectiveness of this family-based approach. From the baseline to the end of the intervention, a significant decrease in participants’ anxiety symptoms was observed, along with medium to large effect sizes for depression symptoms and patients’ IDWG. Given these results, it is possible that a larger sample would increase the chances of detecting statistically significant within-group differences [67].

### Limitations

This pilot study design has some limitations that need to be acknowledged. Patients were recruited only from one dialysis unit, and there was no control group; therefore, caution is needed when using these study data for estimating screening, eligibility, consent, retention, and adherence rates for future large-scale multicenter randomized clinical trials. Moreover, the inclusion criteria were strict, and this led to the exclusion of patients with comorbidities (e.g., cancer) and who had returned to dialysis after kidney transplant failure, which are two very common realities in patients undergoing HD [68,69]. Consequently, this study had a small sample size, which does not allow for estimates of treatment effectiveness due to lack of precision; however, this sample size has been described as reasonable for pilot studies [70]. Lastly, participants were recruited over a two-month period, and therefore, some had to wait for the intervention to start. This waiting time, although short for most, may have caused the dropouts reported in the consent rate, as written consent was re-evaluated one week before the intervention and, at that time, three patients were no longer interested in participating.

## 5. Conclusions

This pilot study represents an important contribution to the ESRD literature, as it suggests that family-based psychoeducational interventions are likely feasible, acceptable, and potentially effective for patients undergoing HD and their family caregivers. Overall, participants appreciated the intervention, which also helped to reduce their psychological distress. The results also highlighted important issues related to recruitment (eligibility and consent rates) and intervention adherence, suggesting that strategies are needed to manage suboptimal rates in order to increase the odds in favor of conducting a large-scale trial. Recruiting multicentric samples and including additional outcome measures would help to build on current findings. In conclusion, research is needed to make family-based psychoeducational interventions more attractive and practical to facilitate implementation in routine dialysis care.

## Figures and Tables

**Table 1:** Content of the intervention program.

Session	Component	Intervention/Aims
1	Supportive	The psychosocial impacts of ESRD and HD on family life: to explore the current beliefs and representations about the disease and treatment and ascertain its negative and positive impacts on family life.
2	Educative	The benefits of physical activity and exercise for patients with ESRD and their family members: to explore the barriers and motivators for exercise and how to integrate physical activity into daily routines.
	Supportive	Emotional regulation: to improve and explore emotion-management strategies by clarifying the interface between emotions, thoughts, and behaviors with examples from experiences with ESRD and HD demands. A guided imagery relaxation technique was also used.
3	Educative	Care with vascular access: to inform about the importance of caring for the vascular access and improve knowledge on the different precautions to be taken with the arteriovenous fistula (before, during, and after each dialysis session). Obs.: The educational component of this session was supported by a nurse with extensive clinical experience in vascular access management in patients undergoing HD.
	Supportive	Interpersonal communication: to provide dyads with communication skills to facilitate interaction with other patients, healthcare professionals, and family members.
4	Educative	Social and community resources: to increase the involvement of families with the community in order to avoid social isolation (by delivering information about patients’ and caregivers’ associations, social events in the community, traveling while on HD, and clarifying patients’ and caregivers’ social rights).
	Supportive	Problem solving: to increase skills to deal with problematic situations associated with ESRD, HD, its side effects, and consequences in daily life (e.g., strategies to increase adherence to poly-medication protocols, to improve memorization, to manage fluids and dietary restrictions in social situations).
5	Educative	Fluids and dietary restrictions in ESRD: to improve dyads’ knowledge about fluids and dietary requirements and how to manage them.
	Supportive	Family identity beyond ESRD: to explore the impacts of chronic disease on family identity; to increase family cohesion through the development of a common identity decentralized from the limitations that ESRD imposes.
6	Supportive	Meaning in life: to encourage the family to identify the different sources of meaning from past and present experiences, reframe the experience with the disease, and establish future life goals. Ritualization and finalization.

ESRD = end-stage renal disease; HD = hemodialysis.

**Table 2:** Participants’ characteristics (*n* = 12).

Participants’ Characteristics	Patients (*n* = 6)	Family Members (*n* = 6)
Gender, *n* (%)		
Male	3 (50%)	0 (0%)
Female	3 (50%)	6 (100%)
Marital Status, *n* (%)		
Single	0 (0%)	1 (16.7%)
Married	5 (83.3%)	5 (83.3%)
Widower	1 (16.7%)	0 (0%)
Employment Situation, *n* (%)		
Employee	1 (16.7%)	3 (50%)
Student	0 (0%)	1 (16.7%)
Housewife	0 (0%)	1 (16.7%)
Retired	5 (83.3%)	1 (16.7%)
Family Relationship, *n* (%)		
Spouse–Partner	3 (50%)	
Parent–Child	3 (50%)	
	Mean (± SD) (Min–Max)	Mean (± SD) (Min–Max)
Age (years)	65.3 (± 13.3) (48–77)	47.3 (± 15.6) (21–67)
Length of time on dialysis (months)	50.7 (± 59.2) (6–153)	

SD = Standard Deviation.

**Table 3:** Results from the Wilcoxon Signed-Ranked Test for pre and post-intervention scores (*n* = 12 participants).

	Pre-Intervention			Post-Intervention					
	Q1	Median	Q3	Q1	Median	Q3	z	*p*	*r*
HADS-Anxiety	3.00	6.50	10.8	1.25	4	7.25	−2.238	0.025	0.646
HADS-Depression	2.25	6.50	9.00	1.00	6.50	8.25	−1.124	0.261	0.325
IDWG (patients only, *n* = 6)	2.18	2.60	3.15	1.63	2.15	3.30	−1.156	0.248	0.472

HADS = Hospital Anxiety and Depression Scale; IDWG = Interdialytic Weight Gain.

## Data Availability

The data that support the findings of this study are available from the corresponding author upon reasonable request.
